# CuFe_2_O_4_ nanoparticles-based electrochemical sensor for sensitive determination of the anticancer drug 5-fluorouracil

**DOI:** 10.5599/admet.1691

**Published:** 2023-03-15

**Authors:** Peyman Mohammadzadeh Jahani, Maedeh Jafari, Farhad Nazari Ravari

**Affiliations:** 1 School of Medicine, Bam University of Medical Sciences, Bam, Iran; 2 Department of Pediatrics, School of Medicine, Kerman University of Medical Sciences, Kerman, Iran; 3 Student Research Committee, School of Medicine, Bam University of Medical Sciences, Bam, Iran

**Keywords:** Modified electrode, 5-fluorouracil, CuFe_2_O_4_ nanoparticles, voltammetriy, electrochemical sensor, SPE

## Abstract

A fast and facile electrochemical sensor for the detection of an important anticancer drug, 5-fluorouracil, is fabricated using CuFe_2_O_4_ nanoparticles modified screen printed graphite electrode (CuFe_2_O_4_ NPs/SPGE). The electrochemical activity of the modified electrode was characterized by chronoamperometry, cyclic voltammetry (CV) and differential pulse voltammetry (DPV) and linear sweep voltammetry (LSV) experiments. The CuFe_2_O_4_ NPs improved the electrochemical properties of the electrodes and enhanced their electroanalytical performance. Electrochemical measurements using differential pulse voltammetry showed a wide linear relationship between 5-fluorouracil concentration and peak height within the range 0.1 to 270.0 μM with a low detection limit (0.03 μM). Further, the sensor was testified with a urine sample and 5-fluorouracil injection sample, and the observed remarkable recovery results replicate its practical applicability.

## Introduction

Cancer is a class of diseases characterized by out-of-control cell growth. Due to ineffective drugs, cancer is the cause of most deaths worldwide. Therefore it's essential to develop cancer research in order to identify causes and develop strategies for prevention, diagnosis, treatments and cure [1,2].

5-Fluorouracil is an antimetabolite fluoropyrimidine analog prescribed as a chemotherapy drug. 5-fluorouracil, a derivative of uracil in which the 5^th^ positioned hydrogen is replaced by fluorine, is used as an excellent antineoplastic agent in treatment of cancer such as colorectal, breast, stomach, pancreatic, and cervical. It acts on cancer cells by directly incorporating into nucleic acids and inhibiting the thymidylate synthase enzyme, which is involved in nucleotide synthesis [3-6].

However, an overdose of 5-Fluorouracil may result in very toxic side effects such as mucositis, leukopenia, nausea, diarrhea, alopecia, neurotoxicity, ocular toxicity, and cardiac toxicity by accumulation. Consequently, controlling the dose of this drug in biological samples and studying the purity of its pharmaceutical forms can help manage its side effects [7-9].

Currently, there are several analytical techniques for the determination of 5-fluorouracil, such as capillary electrophoresis [10], high-performance liquid chromatography [11], Raman spectroscopy [12] and mass spectrometry [13]. In spite of their good performance, these techniques are generally expensive, time-consuming and require relatively complex procedures for sample preparation.

The use of electrochemical sensors presents an alternative to the aforementioned analyses for the determination of 5-Fluorouracil. Electrochemical methods are highlighted and deserve special attention since they present high sensitivity, selectivity, reproducibility, low cost, and rapid response generation, and related to dyes, they present functional groups that can undergo redox reactions, enabling their determination. Among the electrochemical techniques, voltammetry deserves special attention once different sensors (modified or not) can be applied [14-27].

Magnification of the above properties can be achieved by the application of chemically modified electrode. Electrode modification in analytical chemistry has always been an interesting field. Modifiers effectively transport electrons between the electrodes to an analyte. It works by minimizing the over-potential required for the electrode reactions and increasing the electrode's sensitivity and selectivity [28-39].

With the advent of nanoscience, various kinds of nanoparticles are utilized for electrochemical sensors in several analytical methods due to their unique character. Owing to their small size (normally in the range of 1–100 nm), nanoparticles exhibit unique chemical, physical and electronic properties that are different from those of respective bulk materials and can be used to construct novel and improved sensing devices. Compared with the traditional macroelectrodes, nanostructured electrodes show an increased mass-transport rate, a decreased influence of the solution resistance and a higher signal-to-noise ratio [40-54].

In order to develop a miniaturized sensor, screen-printed electrodes (SPEs) are a valuable choice. SPEs produced by printing different inks on plastic or ceramic supports are gaining widespread applicability for the electrochemical monitoring field. Recently, SPEs have emerged as a simple, disposable, nontoxic and low-cost alternative to mercury-based and conventional solid electrodes for the voltammetric determination of many substances. Moreover, they have been shown to be a convenient substrate for nanoparticle modification [55-58].

In the present work, we synthesized CuFe_2_O_4_ nanoparticles (CuFe_2_O_4_ NPs) and screen-printed graphite electrodes were modified with CuFe_2_O_4_ NPs using the drop-casting method. The resulting modified electrode is applied to the determination of 5-fluorouracil by differential pulse voltammetry.

## Experimental

### Apparatus and chemicals

All the electrochemical measurements were carried out on a PGSTAT302N potentiostat/galvanostat Autolab. The measurement cell consisted of SPGE (DropSens; DRP-110: Spain) containing a graphite counter electrode, a graphite working electrode, and a silver pseudo-reference electrode. Solution pH values were determined using a 713 pH meter combined with a glass electrode (Metrohm, Switzerland). 5-fluorouracil and all chemicals used were of analytical grade and were used as received without any further purification and were obtained from Merck and Sigma Aldrich. Orthophosphoric acid was utilized to prepare the phosphate buffer solutions (PBSs), and sodium hydroxide was responsible for adjusting the desired pH values (pH range between 2 and 9).

### Preparation of modified electrode

CuFe_2_O_4_ NPs/SPGEs were prepared by modifying the bare working electrode of an SPGE using the drop-casting method. Briefly, 4 μL of the solution of CuFe_2_O_4_ NPs (1 mg/mL) were dropped onto the working electrode surface and dried at room temperature. The obtained electrode was noted as CuFe_2_O_4_ NPs/SPGE.

The surface areas of the CuFe_2_O_4_ NPs/SPGEs and the un-modified SPGEwere obtained by CV using 1 mM K_3_Fe(CN)_6_ at various scan rates. Using the Randles–Sevcik equation for CuFe_2_O_4_ NPs/SPGEs, the electrode surface was found to be 0.113 cm^2^ which was about 3.6 times greater than un-modified SPE.

## Results and discussion

### Electrochemical behavior of 5-fluorouracil at the surface of various electrodes

The effect of the electrolyte pH on the oxidation of 50.0 μM 5-fluorouracil was investigated at CuFe_2_O_4_ NPs/SPGE using differential pulse voltammetry (DPV) measurements in the PBS in the pH range from 2.0 to 9.0. According to the results, the oxidation peak current of 5-fluorouracil depends on the pH value and increases with increasing pH until it reaches the maximum at pH 7.0 and then decreases at higher pH values. The optimized pH corresponding to the higher peak current was 7.0, indicating that protons are involved in the reaction of 5-fluorouracil oxidation.

**Figure 1.**
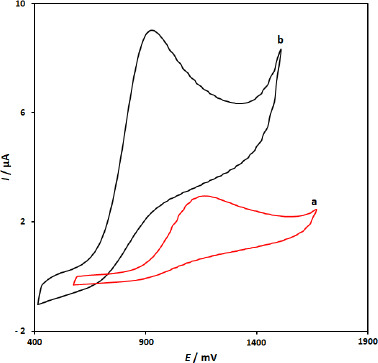
Cyclic voltammograms of a) bare SPGE, b) CuFe_2_O_4_ NPs/SPGE in the presence of 100.0 μM 5-fluorouracil in 0.1 M phosphate buffer solution, pH 7.0.

Figure 1 displays CV responses from the electrochemical oxidation of 100.0 μM 5-fluorouracil at the surface of CuFe_2_O_4_ NPs/SPGE (curve b) and bare SPGE (curve a). The results showed that the oxidation of 5-fluorouracil is very weak on the surface of the bare SPGE, but the presence of CuFe_2_O_4_ NPs in SPGE could enhance the peak current and decrease the oxidation potential (decreasing the overpotential). A substantial negative shift of the currents starting from oxidation potential for 5-fluorouracil and a dramatic increase of the current indicates the catalytic ability of CuFe_2_O_4_ NPs/SPGE to 5-fluorouracil oxidation. The results showed that the using of CuFe_2_O_4_ nanoparticles (curve b) definitely improved the characteristics of 5-fluorouracil oxidation, which was partly due to excellent characteristics of CuFe_2_O_4_ NPs, such as good electrical conductivity and high chemical stability.

### Effect of scan rate on the determination of 5-fluorouracil at CuFe_2_O_4_ NPs/SPGE

**Figure 2.**
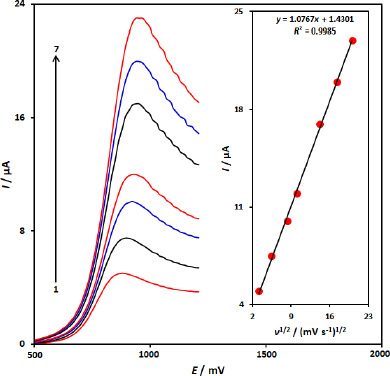
Linear sweep voltammograms of 5-fluorouracil at CuFe_2_O_4_ NPs/SPGE at different scan rates, 1-7 correspond to 10, 30, 70, 100, 200, 300 and 400 mV s^-1^ in 0.1 M phosphate buffer solution, pH 7.0. Inset shows a plot of *I*_pa_ versus *ν*^1/2^ for the oxidation of 5-fluorouracil at CuFe_2_O_4_ NPs/SPGE.

The influence of potential scan rate (*ν*) on *I*p of 100.0 μM 5-fluorouracil at the CuFe_2_O_4_ NPs/SPGE was studied by linear sweep voltammetry (LSV) at various sweep rates (Figure 2). As shown in Figure 2, the peak currents of 5-fluorouracil grow with the increasing scan rates and there are good linear relationships between the peak currents and *ν*^1/2^ (square root of scan rate) (Figure 2 inset). The regression equation is *I*_pa_ = 1.0767 *ν*^1/2^ +1.4301 (*I*_pa_/ μA, *ν* / mV s^-1^, *R*^2^= 0.9985), indicating the oxidation process of 100.0 μM 5-fluorouracil at the CuFe_2_O_4_ NPs/SPGE was diffusion-controlled.

**Figure 3.**
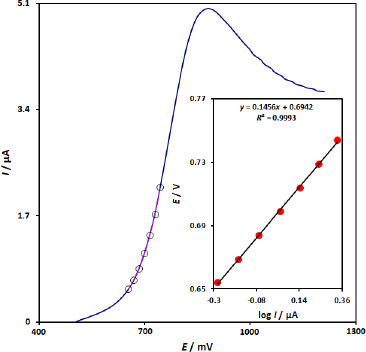
Linear sweep voltammograms response for 100.0 μM 5-fluorouracil with 10 mV s^-1^ scan rate. Inset: The Tafel plot derived from the rising part of the corresponding voltammogram

To obtain further information on the rate-determining step, the Tafel plot for oxidation of 100.0 μM 5-fluorouracil at the surface of CuFe_2_O_4_ NPs/SPGE using the data derived from the raising part of the current–voltage curve has been recorded in Figure 3. Using the slope of the Tafel plot at a scan rate of 10 mV s^-1^, the value of electron transfers coefficient (*α*) was determined as 0.6, confirming an irreversible process for the oxidation of 5-fluorouracil on the surface of CuFe_2_O_4_ NPs/SPGE.

### Chronoamperometric studies

**Figure 4.**
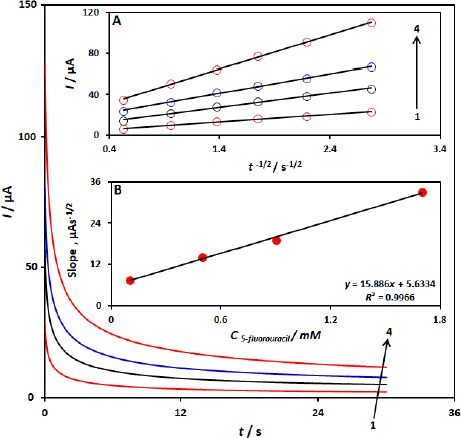
Chronoamperograms obtained at the CuFe_2_O_4_ NPs/SPGE in 0.1 M phosphate buffer solution, pH 7.0, for different concentrations of 5-fluorouracil. The 1-4 correspond to 0.1, 0.5, 0.9 and 1.7 mM of 5-fluorouracil. (A) Plots of *I vs. t*^-1/2^ for electrooxidation of 5-fluorouracil obtained from chronoamperometry. (B) Plot of the slope of the straight lines against 5-fluorouracil concentration.

The electrochemical oxidation of 5-fluorouracil by a CuFe_2_O_4_ NPs/SPGE was also studied by chronoamperometry. Chronoamperometric measurements of different concentrations of 5-fluorouracil at CuFe_2_O_4_ NPs/SPGE were done by setting the working electrode potential at 1000 mV (Figure 4). In chronoamperometric studies, we have determined the diffusion coefficient, *D*, of 5-fluorouracil. The experimental plots of *I* versus *t*^-1/2^ with the best fits for different concentrations of 5-fluorouracil were employed (Figure 4 A). The slopes of the resulting straight lines were then plotted versus the 5-fluorouracil -concentrations (Figure 4 B), from whose slope and using the Cottrell equation (1):


(1)

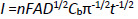



We calculated a diffusion coefficient of 8.3×10^-5^ cm^2^ s^-1^ for 5-fluorouracil.

### Calibration curve and limit of detection

**Figure 5.**
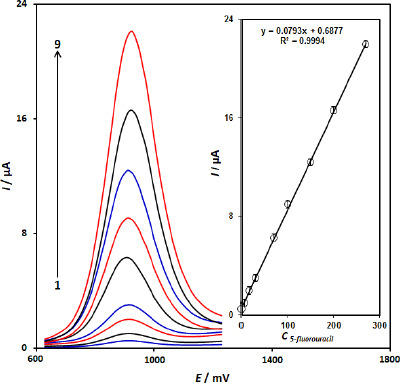
Differential pulse voltammograms of the CuFe_2_O_4_ NPs/SPGE in 0.1 M phosphate buffer solution (pH7.0) containing different concentrations of 5-fluorouracil, Numbers 1–9 correspond to 0.1, 5.0, 15.0,30.0, 70.0, 100.0, 150.0, 200.0 and 270.0 μM of 5-fluorouracil. (B) the plot of the voltammetric peak current as a function of 5-fluorouracil concentration.

Since DPV has a much higher current sensitivity than cyclic voltammetry, we used the DPV method for the determination of 5-fluorouracil (Step potential=0.01 V and pulse amplitude=0.025 V). Figure 5 shows DPVs of different concentrations of 5-fluorouracil and the obtained calibration curve. The results showed a linear segment for 5-fluorouracil concentration from 0.1 to 270.0 μM 5-fluorouracil (Figure 5), with a regression equation of *I*_p_ = 0.0793*C*_5-fluorouracil_ + 0.6877 (*R*^2^= 0.9994, n=9). The detection limit, LOD, was obtained by using the equation (2):


(2)

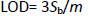



where *S*_b_ is the standard deviation of the blank response (n=15) and *m* is the slope of the calibration plot. The limit of detection was determined to be 0.03 μM for 5-fluorouracil.

### Real sample analysis

To investigate the applicability of the proposed sensor for the voltammetric determination of 5-fluorouracil in real samples, we selected urine and 5-fluorouracil injection samples for the analysis of 5-fluorouracil contents. The 5-fluorouracil contents were measured after sample preparation using the standard addition method. The results are given in Table 1. According to the table, the recovery values within 98.0-103.6 % confirm the powerful ability of CuFe_2_O_4_ NPs/SPGE for the determination of 5-fluorouracil in real samples.

## Conclusions

The fabrication of sensors for the measurement of 5-fluorouracil was achieved using screen-printed graphite electrodes modified with CuFe_2_O_4_ NPs. The CuFe_2_O_4_ NPs remarkably decreased overvoltage and improved the electrochemical response of 5-fluorouracil in terms of specificity, sensitivity and current response. Under optimized conditions, differential pulse voltammetry exhibited linear dynamic ranges from 0.1-270.0 μM with a detection limit of 0.03 μM. Also the CuFe_2_O_4_ NPs/SPGE was used to detect 5-fluorouracil in real samples and produced satisfactory results.

## Figures and Tables

**Table 1.** The application of CuFe_2_O_4_ NPs/SPGE for determination of 5-fluorouracil in real samples (n=3)

Sample	*C* / μM		Recovery, %	RSD, %
	Spiked	Found		
Urine	0	-	-	-
	5.0	4.9	98.0	1.9
	7.0	7.2	102.9	3.0
5-fluorouracil injection	0	3.0	-	3.2
	2.5	5.7	103.6	1.6
	4.5	7.4	98.7	2.8
